# Sugar-Coated: Can Multivalent Glycoconjugates Improve upon Nature’s Design?

**DOI:** 10.1021/jacs.4c08818

**Published:** 2024-09-28

**Authors:** Kathryn G. Leslie, Sian S. Berry, Gavin J. Miller, Clare S. Mahon

**Affiliations:** † Department of Chemistry, Durham University, Durham DH1 3LE, United Kingdom; § Centre for Glycoscience and School of Chemical and Physical Sciences, Keele University, Keele, Staffordshire ST5 5BG, United Kingdom

## Abstract

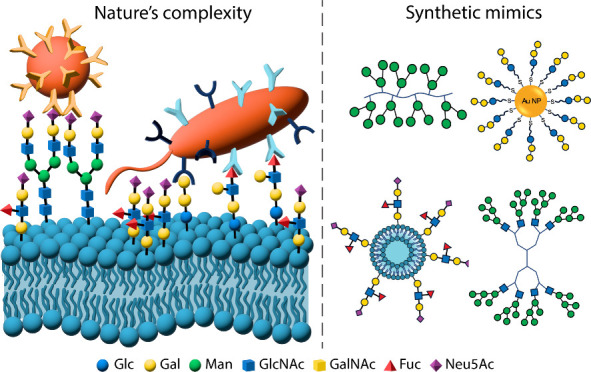

Multivalent interactions between receptors and glycans play an important role in many different biological processes, including pathogen infection, self-recognition, and the immune response. The growth in the number of tools and techniques toward the assembly of multivalent glycoconjugates means it is possible to create synthetic systems that more and more closely resemble the diversity and complexity we observe in nature. In this Perspective we present the background to the recognition and binding enabled by multivalent interactions in nature, and discuss the strategies used to construct synthetic glycoconjugate equivalents. We highlight key discoveries and the current state of the art in their applications to glycan arrays, vaccines, and other therapeutic and diagnostic tools, with an outlook toward some areas we believe are of most interest for future work in this area.

## Introduction

**Figure 1:**
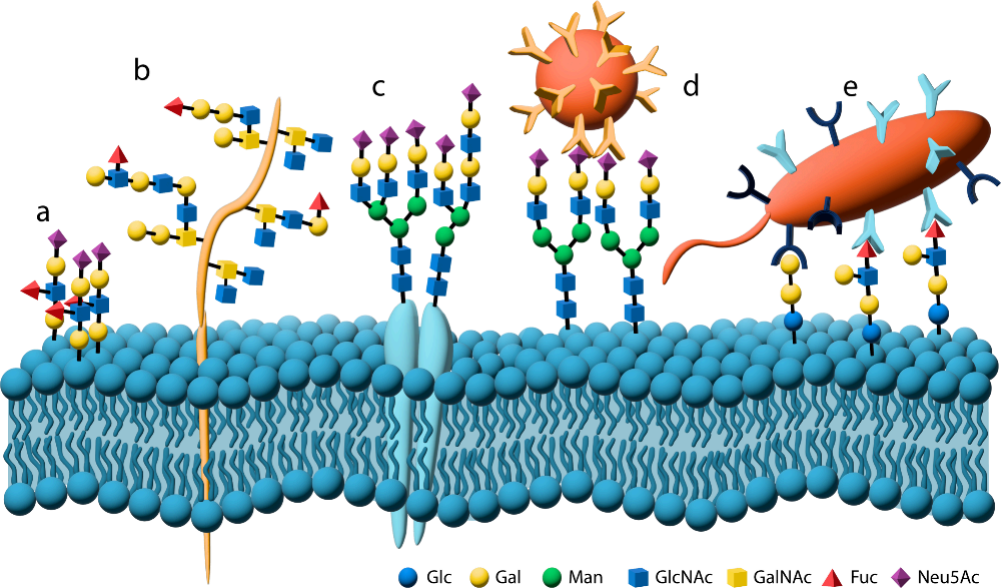
A representation of the diversity in form and function of the glycocalyx. a) Glycolipids, for example the glycosphingolipid sialyl Lewis x antigen shown; b) proteoglycans, for example the mucin MUC1, displaying branched *O*-linked glycans which contribute to an epithelial cell’s protective barrier;^7^ c) membrane-bound glycoproteins, for example the depicted major histocompatibility complex II, decorated with *N*-linked glycans implicated in antigen binding;^8^ d) viral recognition of the mammalian cell surface, for example influenza binding sialic acid terminal residues through surface hemeagglutinin;^9^ e) bacterial recognition of the mammalian cell surface, for example *Pseudomonas aeruginosa* proteins LecA and LecB binding d-galactose-terminated Gb3 and l-fucose-terminated Lewis a glycans, respectively.^10^

Carbohydrate recognition plays a pivotal role in many different biological processes, including cell–cell recognition, pathogenesis and the immune response. The mammalian cell surface is decorated with a rich array of complex glycans tethered to membrane-bound proteins^1^ and lipids,^2^ forming a layer called the glycocalyx (Figure 1). The multivalent presentation of these glycans is crucial in the mediation of self-recognition and immune processes,^3^ with cell surface glycans recognized by viral and bacterial pathogens during adhesion and infection events (Figure 1d, e).^4^ Variation in the composition of cell surface glycans has also been implicated in many disease states—hypersialylation and fucosylation are broadly associated with evading immune response in many types of cancer,^5^ for example, and a reduction in the complexity of the glycocalyx has been implicated in neurodegenerative disease states.^6^

Often these key processes of physiology and pathology rely on interactions with multiple copies of a displayed glycan, and this multivalent presentation furnishes selectivity and avidity not achievable with individual (or monovalent) interactions. The attractiveness of this precise, yet tunable molecular recognition has led to the design of biomimetic systems which exploit the power of multivalent glycan interactions, for applications in drug discovery, vaccines, diagnostics, and tools to probe fundamental biological interactions. Despite significant progress, however, we remain far from achieving the complexity and intricacy displayed in nature.

This perspective will briefly explore the basis of the potent, selective yet dynamic recognition and binding enabled by multivalent glycan presentation, and then introduce the scaffolds commonly utilized to produce synthetic equivalents. We will highlight key examples of synthetic systems which harness multivalent glycan interactions in the context of developing diagnostics, designing vaccines and therapeutics, and probing biological recognition through glycan arrays. Finally, we will discuss the outlook for this exciting field, exploring the key opportunities and challenges presented.

## Multivalent Recognition of Carbohydrates

**Figure 2:**
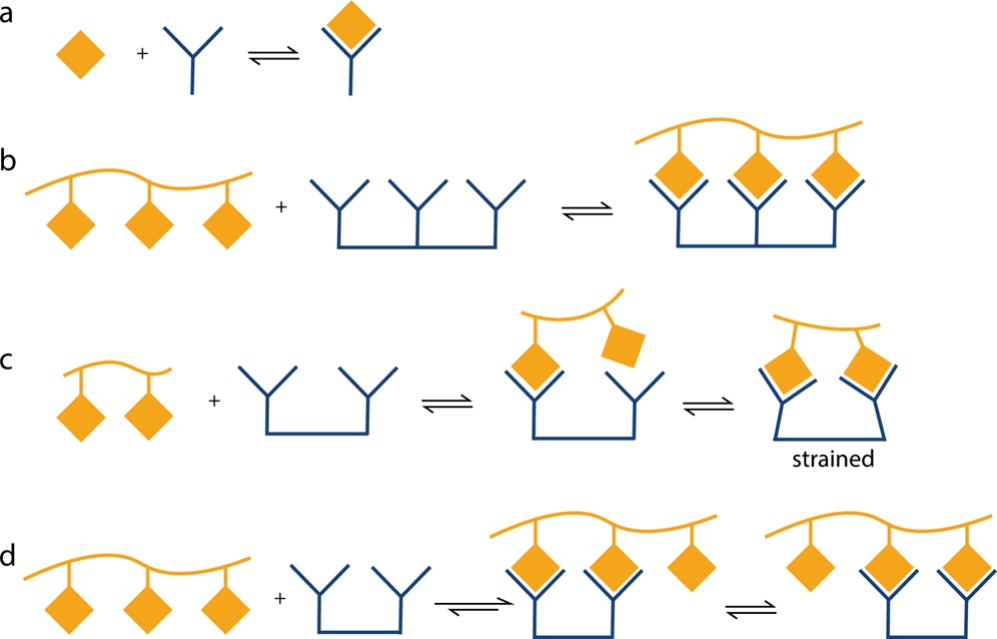
Representations of a) monovalent binding; b) multivalent binding; c) reduced enthalpy due to conformational strain associated with multivalent binding and nonoptimized linker length; and d) impact of increasing the effective local concentration through multivalent ligand presentation.

It has long been established that multivalent presentation of recognition motifs can enable increases in affinity and avidity of recognition events (Figure 2a,b).^11−13^ Affinity describes the strength of an individual binding interaction, while avidity describes the accumulated strength of constituent individual recognition events. Multivalent binding can increase the selectivities and affinities of monovalent interactions by many orders of magnitude, far exceeding additive effects. This strategy is employed consistently and successfully in biological systems, across different classes of biological macromolecules, including in protein–DNA,^14^ protein–RNA,^15^ protein–protein,^16^ and protein–carbohydrate interactions.^11^ For example, heterodimeric binding of different transcription factor proteins to DNA allows for precise, combinatorial-mediated regulation of transcription.^17,18^ Furthermore, the usual low-affinity micromolar recognition of 3–5 nucleotide sequences displayed by RNA-binding proteins^19^ are enhanced through multivalency to interactions of much higher avidities.^20^ Multivalent protein–protein interactions are frequent features of signaling complexes which combine the functions of several proteins in a cascade by binding to a central scaffold.^16,21,22^

Unlike proteins and nucleic acids, however, complex carbohydrates are not created using a templated biosynthesis, and confer macromolecules with far more structural diversity, owing to their large monomer palette, multiple attachment points that allow for branching, and variable enzymatic modification.^23^ Glycan presentation on biological interfaces is therefore often heterogeneous, complicating the investigation of structure to function relationships.^24^ However, advances in glycan synthesis and characterization tools, such as glycoprotein enrichment, mass spectrometry,^25^ microarrays, chromatography, and informatics, are allowing for rapid strides in increasing our understanding of glycobiology.^26,27^

Lectins are proteins which bind carbohydrates, and are often complexed as multimers possessing several recognition sites, with the ability to interact with multiple copies of the relevant glycan. The idea that polydentate ligands could form more stable complexes than their equivalent monodentate ligands (the chelate effect) is a concept that has long been familiar to supramolecular chemists.^28^ However, it was not until seminal work by Whitesides and co-workers demonstrated that polyacrylamides with pendant sialosides were potent inhibitors of the agglutination of red blood cells by the influenza virus,^29^ that this concept became accepted hegemony within the study of higher-order biochemical systems. The inhibition of hemeagglutination assay was an early method used to determine the extent of carbohydrate binding to a lectin. This was achieved by measuring the concentration of the competing monovalent carbohydrate required to prevent the aggregation of red blood cells by influenza, which is facilitated by the interaction between sialic acid residues on the surface of the red blood cell and influenza surface hemagglutinin.^30^ In this and subsequent work,^31,32^ it was shown that a drastic improvement on the inhibitory effect of monosialic acid derivatives was possible using polymers bearing 10s of sialic acid groups. Inhibition of agglutination was proposed to be a consequence of the entropic favorability of multivalent binding, and steric blocking of the interaction between bound virus particles and red blood cells. The inhibitory potency of these polymers bearing multiple copies of randomly displayed binding motifs was 10^4^–10^5^ times higher than the α-methyl sialoside monomer.^31^ This effect, when coupled with rational design of the orientation and spatial arrangement of binding motifs such as displayed by the STARFISH dendrimer,^33^ which incorporated 5 trisaccharide binding motifs aligned with the pentameric Shiga toxin binding sites, can provide 10^7^-fold enhancement. The so-termed cluster glycoside effect has since been effectively demonstrated across a range of synthetic and biological systems, with broad-reaching implications in drug and diagnostic design, as well as furthering fundamental understanding of biological recognition processes.^34−39^

The favorable free energy changes of multivalent binding interactions are driven by the enthalpic and entropic contributions of linked carbohydrate ligands with a receptor protein. A favorable binding enthalpy is driven by networks of hydrogen bonds between the carbohydrate ring hydroxyl groups and amino acid residues,^40^ and the C–H bond interactions with typically aryl residue-rich binding pockets.^41^ The binding site is often stabilized, or further positive interactions facilitated, by incorporation of metal ions, such as Ca^2+^ and Mn^2+^.^42^ Higher affinity lectin-carbohydrate interactions correlate with structures that display “subsite multivalency”, i.e., multiple distinct regions that can bind the constituent monosaccharides within a complex glycan.^43^ In multivalent interactions, the enthalpy contribution is not necessarily the sum of constituent monovalent interactions, as a second binding event could cause ligand or linker conformational strain, or distort the geometry of binding site contacts, lowering the overall combined enthalpy (e.g., Figure 2c). From an entropic perspective, there are several factors that influence any binding event, including the conformational entropic penalty and the added disorder of solvent molecules displaced into the bulk. In the first binding event of a multivalent system, however, there is also the reduction of rotational and translation freedom as two molecules are effectively reduced to a single species. As this penalty does not apply again to subsequent binding events, these are referred to as entropically enhanced.^44^

The influence of a previous binding event upon the enthalpy and entropy (and therefore free energy) of a subsequent binding eventis known as cooperativity. Cooperativity is quantified with a parameter α, which is >1 when cooperativity is synergistic, <1 when cooperativity is negative and interfering, and =1 when recognition is noncooperative/additive. The classic biochemical example of positive cooperativity is the allosteric binding of oxygen to hemoglobin,^45^ a tetrameric protein which binds four oxygen molecules, with increases in affinity for each binding event as a result of allosteric effects upon the quaternary structure of the protein.^46^ For example, modest positive cooperativity has been demonstrated for multivalent glycan binding interactions with the cholera toxin,^47−49^ which has a pentameric subunit that binds to multiple copies of GM1 glycolipid on the cell surface,^50^ before undergoing endocytosis. Many multivalent binding systems in fact display negative cooperativity, which, along with fast *k*_on_/*k*_off_ rates,^51^ is thought to contribute to the dynamic nature of binding in many biological multivalent interaction systems.^52^

Even in systems where subsequent binding is negatively cooperative, multivalent systems still provide useful degrees of higher-avidity binding.^53^ In addition to the above-mentioned contributions, the “statistical effect” ^54^ describes the impact of having multiple ligands present near a binding site, equivalent to increasing the local concentration of a ligand such that after any dissociation, reassociation is highly favored (Figure 2d).^12^ This effectively lowers the rate of ligand dissociation (*k*_off_) rather than impacting the rate of binding (*k*_on_).^55,56^ For example, it has been shown^57^ that a bivalent IgG antibody and a monovalent fragment have similar association rates for binding to bacterial cells, but the enhanced overall avidity of the bivalent antibody is due to the 40 times slower rate of dissociation. High local concentrations of clustered glycolipids have also been shown to induce a 9-fold increase in the rates of enzymatic galactosylation,^58^ reminiscent of this same statistical effect. The emerging idea of “superselective” recognition^59^ by multivalent glycoconjugates may be satisfied using low affinity interactions, through a combination of mutivalency and combinatorial entropy effects.

Many carbohydrate–lectin interactions are multivalent with respect to both the carbohydrate and the lectin recognition domain, furnishing access to multiple binding modes. Multivalent glycoconjugates may bind to multiple recognition sites on a single lectin, or cross-link recognition sites on adjacent lectins. It is often difficult to deconvolute the contribution of these distinct binding modes, particularly in complex biological systems, yet their impacts may be profound, as has been demonstrated by a model system exploring recognition of DC-SIGN.^60^ Mannosylated quantum dots were observed to bind both DC-SIGN, a cell-surface lectin with four carbohydrate recognition sites, and DC-SIGNR, a related lectin which can participate in cross-linking on account of the orientation of recognition domains. Recognition of both lectins proceeds via an enthalpy-driven process with nanomolar *K*_d_. DC-SIGN recognition proceeded with a smaller entropic penalty and subsequently lower *K*_d_, as may be expected. FRET studies demonstrated that DC-SIGN recognition displayed a single second order *k*_on_, but DC-SIGNR recognition proceeded via rapid initial binding followed by a slower secondary interaction, highlighting the complexities of multivalent recognition.

There are several suggested reasons for why nature employs the multivalent presentation of low-affinity interactions, rather than many different, specific and high-affinity contacts. First, multivalency provides a chance to increase binding affinity over a dynamic range, giving a breadth of response to mono or multivalent ligands, rather than a simple on/off switch.^61^ Second, it has been hypothesized that there is an evolutionary efficiency to utilizing existing interactions rather than constructing new ones.^11^ When used in a combinatorial fashion, several new binding motifs with varying affinities could be differentiated by a signaling system containing a small set of monovalent interactions, as described above for the case of transcription factors.^17,18^

Finally, multivalent interactions allow for recognition events and aggregate structures over large surface areas. For example, it is thought they may play a role in processes such as cell–cell signaling and tissue structure at the membrane^62^ and glycan-protein aggregates on the cell surface have been shown to provide a scaffold for phase domain separation that mediates receptor ligand enrichment and therefore signaling transduction.^63^

## Synthetic Approaches to Create Multivalent Glycoconjugates

**Figure 3:**
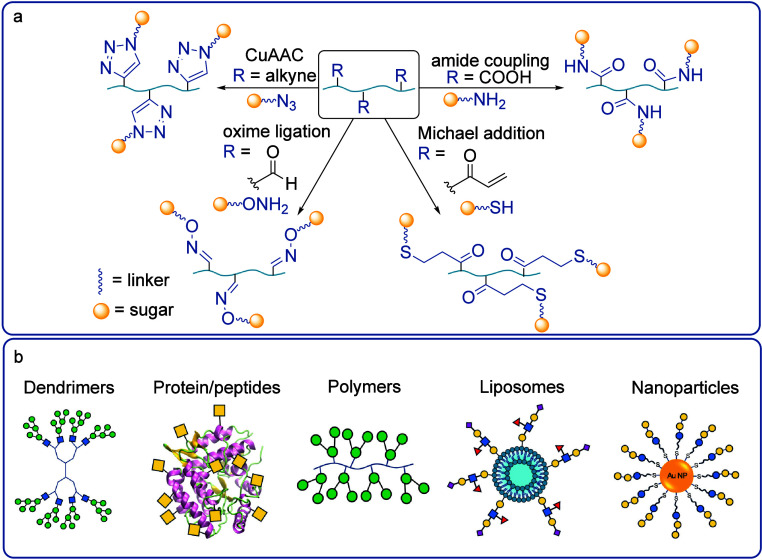
a) Common attachment methods for the synthesis of multivalent glycoconjugates. b) Classification of constructs commonly used to achieve multivalent glycan presentation.

The synthesis of multivalent systems is typically achieved by conjugation of an appropriately compatible glycan onto a central or core structure suitably equipped for multiple points of glycan attachment. Robust chemistries, such as copper-catalyzed azide–alkyne cycloaddition (CuAAC), amide coupling, Michael addition and oxime or hydrazone ligation are frequently used as attachment methods (Figure 3a).^64^ Using this approach to construction, linkers are often employed to furnish the sugar with the desired functional group for attachment. For example, azides are often used at the terminus of a pendant alkyl chain attached to the anomeric center of the sugar. The length of the linker and its hydrophilic–hydrophobic balance are important considerations, with highly hydrophilic examples such as polyethylene glycol (PEG) and amides often favored.^65^ Longer chain linkers allow for increased flexibility in the system which facilitates multivalent interactions through an increase in binding probability. However, in some cases this must be tensioned against the entropic penalty for loss of flexibility upon binding,^66^ although thermodynamic models do indicate free energy is only weakly dependent on the conformational penalty of flexible linkers.^67^ The length of the linker is also dependent on the target; for example, if the recognition domain lies within a deep binding pocket, a longer linker is required compared to a system with a shallow binding pocket,^68^ and in order to achieve high-affinity, selective recognition, a bespoke consideration of the target system is required.

In order to facilitate multivalent binding, surfaces or macromolecular scaffolds are often used to present glycans in two or three dimensions, with a high degree of diversity in the structures used (Figure 3b).^64,69,70^ Dendrimers present highly structurally defined systems,^71^ allowing chemists to know the exact multivalency of their system, however they often require significant synthetic effort to construct, and the byproducts of incomplete reactions can be difficult to separate. Cyclic peptides are similarly well-defined with regards to their positional definition for sugar attachment and ultimate multivalency^72^ allowing for a degree of preorganization within receptor design. Moving toward larger polypeptide scaffolds such as proteins imparts benefits including biocompatibility and greater surface areas, while maintaining defined site modification through chemoselective ligation methods.^73^ Polymeric scaffolds allow for a wide diversity in their macrostructures and therefore the geometry of glycan presentations, with linear, self-assembled micellar, nanofiber, and surface-grafted glycoconjugates reported.^74^ The facile production of self-assembled systems such as micelles and liposomes make them attractive scaffolds, and although the glycan density can be more challenging to monitor, the geometric fluidity of embedded glycans can be ideal for some applications.^65^ Heteromultivalent systems, which display mixtures of different glycans, are still an under-investigated area compared to systems displaying multiple copies of a single glycan.^75^ Strategies toward their synthesis include stepwise solid phase synthesis of oligonucleotides^76,77^ or peptide-like molecules with “clickable” side-chains,^78,79^ controlling equivalents on dendrimer scaffolds,^80^ as well as a variety of orthogonal reaction strategies.^81^ Self-assembled scaffolds such as micelles and liposomes provide a simple route to the incorporation of different carbohydrates without the need for bottom-up orthogonal chemistries.^82,83^

The presentation of multiple carbohydrate recognition motifs on a synthetic macromolecular scaffold also allows for the use of simplified carbohydrate structures,^84^ the inclusion of secondary recognition motifs,^85^ and variation of glycan density. The design of receptors is not always entirely rationalized, however, with studies highlighting complexities in the relationships between carbohydrate density and inhibitory potency.^68^ The use of polymer or nanoparticle scaffolds can also confer multivalent receptors with additional functionality such as response to environmental stimuli including temperature, allowing for control over carbohydrate presentation and subsequent recognition behavior.^86−88^

A key development in the pursuit of complex synthetic multivalent systems was in the automated production of the glycan itself. Automated glycan assembly (AGA) was pioneered by Seeberger in the early 2000s^89^ and has greatly simplified access to complex glycans by moving the synthesis onto a solid phase support.^90^ Compared to automated, on-surface polypeptide or polynucleotide synthesis, oligosaccharide synthesis presents several challenges. Each monomer has multiple potential sites of attachment, with the opportunity for branched, rather than only linear, structures.^91^ Glycosylation creates new stereogenic centers, and so protecting group/building block and glycosylation methodology considerations to ensure competent regio- and stereoselectivity are essential but complex. Additionally, there are hundreds of known monosaccharide building blocks, in contrast to the limited palette of amino acids and nucleosides. AGA has progressed many of these issues, by developing solid phase supports to construct oligosaccharides from the reducing end to the nonreducing end. The solid support allows for facile washing of excess reagents between deprotection and coupling steps, and steps can be programmed in a fully automated manner.^92^ AGA now allows access to 100-mer mannans,^93,94^ for example, and the methods and protecting groups strategies have grown to allow the incorporation of some more challenging targets such as highly sulfated structures,^95,96^ multiple *cis* glycosidic linkages,^97^ and the incorporation of 2-deoxy-2-fluoro-sugars as labels.^98^ One of the remaining restrictions to the synthesis of more complex glycans is now the efficient and scalable synthesis of more unusual monosaccharide building blocks, and the specifics of a unique oligosaccharide’s final “global deprotection” steps once cleaved from the solid support.^99^

Chemoenzymatic synthesis, where synthetic building blocks are assembled or modified by enzymes, is another strategy which has pushed forward the production of more complex glycans.^100−102^ This process has similarly been automated, using sulfonated tags that allow for postenzymatic solid phase extraction of product.^103^ Orthogonal, five-protecting-group strategies have allowed the elaboration of a core pentasaccharide that is common to eukaryotic *N*-linked glycans, to furnish complex libraries of branched oligosaccharides.^104^ The development of automated and enzymatic methods in the past 20 years is starting to move the synthesis of complex glycans from the domain of a few expert laboratories into more widely employed and commercially accessible methods, increasing the range of glycans available, and eliminating a key barrier to the development of more complex multivalent glycoconjugates.^105^

## Glycan Arrays

**Figure 4:**
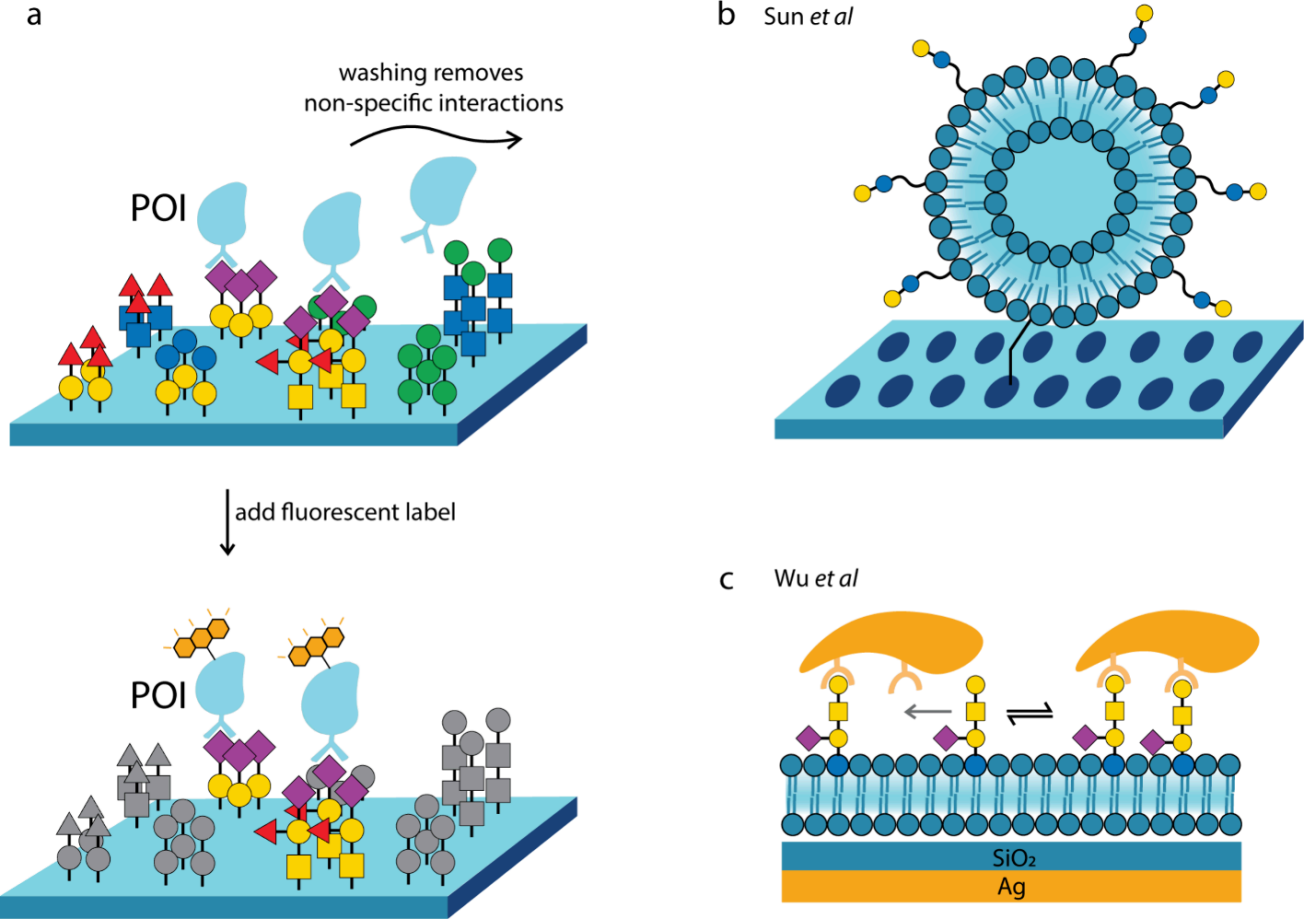
Schematics of a) typical glycan array setup for investigating the glycan-binding preferences of a protein of interest (POI); b) spherical liposome-based 3D glycan array;^144^ and c) supported lipid bilayer-based glycan array.^146^

Glycan arrays are a high-throughput approach to screen glycan recognition capabilities against target proteins or other binding partners of interest. This technology was pioneered in the early 2000s, with the noncovalent deposition of glycolipids onto nitrocellulose surfaces^106^ or glass slides,^107^ furnishing two-dimensional platforms that allow the routine profiling of protein–carbohydrate interactions. Once the protein of interest has been incubated with the slides containing the immobilized glycan, a washing cycle removes any nonspecific binding before further incubation with a labeling fluorophore enables read out from competent ligands regarding their apparent binding affinity (Figure 4a).^108^ This technology is now well established,^109,110^ and preprinted microarrays can be purchased with hundreds of defined saccharides attached to the surface.

Development of orthogonal attachment chemistries means that sugars can be grafted onto distinct areas of a given solid support using a myriad of methods, including thiol–ene and tetrazine click reactions, imine condensations, and nonspecific photolabeling strategies.^111^ These methods, along with noncovalent attachment strategies such as biotin–streptavidin and oligonucleotide binding, or hydrophobic or electrostatic interactions with nitrocellulose and glass surfaces, have been thoroughly reviewed elsewhere.^111−113^

Glycan arrays have aided in the discovery of many interactions of glycans and binding proteins from viruses,^114−116^ bacteria,^117^ and parasites.^118,119^ They have also been utilized to elucidate many glycan–immune protein interactions,^120^ and as screening tools for antiglycan antibody biomarkers.^121^ Notably, glycan arrays aided in the discovery of significant glycan–glycan interactions which were previously thought to have much lower avidities than glycan–protein interactions.^122^ Array studies showed that the lipo-oligosaccharides (LOS) and lipopolysaccharides (LPS), which are found in the outer membrane of Gram-negative bacteria,^123^ can bind to numerous host cell glycans with dissociation constants comparable to lectins. Surface plasmon resonance (SPR) analysis indicated that the dissociation constants for the interactions between *Haemophilus influenzae* LOS/LPSs and the Lewis a antigen, for example, ranged from 0.1 to 10 mM.^122^ The sensitivity of glycan arrays can be enhanced in some cases through the use of oligomeric forms of the protein of interest,^124^ exploiting the cluster glycoside effect with respect to both binding partners. A reverse technology to glycan arrays is also available, with the lectin component printed onto a solid support, and a glycan or glycoprotein used as the incubation partner. This technique is commonly used to investigate changes in protein glycosylation.^108,125,126^

One advantage of glycan arrays is the very small amount of material that is needed for screening (down to femtograms of glycan), and which is printed onto slides with automated robots that can routinely print spots with 100 μm diameters,^127^ or down to 1 μm with tip-based lithography and photochemical surface attachment.^128^ However, despite the small amounts required, the total synthesis of complex oligosaccharides incurs a high cost and time penalty and in some respects remains a bottleneck to accessing the plethora of known (and required) structures for exploratory biology, despite the advances in synthetic techniques discussed above.^92^

The spacing and orientation of glycans on a biological interface such as the cell surface is critical for carbohydrate recognition, in particular for multivalent binding.^129^ However, this spatial orientation can be difficult to control or emulate with any labeling or attachment strategy in two dimensions.^130^ Accordingly, there have been attempts to produce glycan microarray technologies more representative of the three-dimensional fluidic membrane presentation of carbohydrates in biology.^131,132^ By utilizing larger or branching scaffolds (e.g., Figure 3b), which are themselves attached to a solid support, a more flexible binding environment can be introduced. Dendrimers,^133^ polymers,^134−136^ nanoparticles,^137^ proteins^138^ (for example mucins),^139^ nucleic acids,^77,140,141^ and coordination cages^142^ have all been utilized to this effect, and can offer finer control of overall glycan density, a degree of further flexibility in glycan presentation. There is often, however, a trade-off between incorporating enough flexibility to allow multivalent binding without steric hindrance and having defined and complete knowledge of the geometry of glycan presentation, such that information about spacing or extent of binding can be accurately inferred.

One interesting route to overcoming the enforced structural presentation of surface attachment, and to introduce equilibrium control over density and orientation, is the modification of glycan array approaches to incorporate fluid membranes, for example by using liposomes or lipid bilayers. For example, by incorporating varying amounts of D-mannose-appended glycolipids into a supported lipid bilayer, Guo and co-workers have showed *Escherichia coli* FimH adhesion progressing from mono- to multivalent binding as the density of mannosyl groups in the membrane increased.^143^ Sun and co-workers have produced biotinylated liposomes which can be attached to streptavidin-coated slides (Figure 4b), and then further functionalized with lactosides via a Staudinger ligation. These systems showed competent binding with β-d-galactose binding lectin,^144^ and in a later study incorporation of GM1 and GM3 gangliosides into similar systems allowed the discrimination of a panel of four lectins.^145^

By allowing for the free diffusion of glycans, these membrane-based systems can equilibrate with glycans clustered at the correct distances and orientations for optimal multivalent binding. Separately, this concept has also been demonstrated in supported lipid bilayers (Figure 4c), and combined with SPR in a nanocube technology to deliver quantitative binding information.^146^ A particular advantage of membrane-based systems is that heteromultivalency can be explored without the need to synthesize glycoconjugates with specific presentations of different glycans. Instead, a mixture of ligands can be incorporated into the membrane in different ratios and the dynamic nature of the system allows the observation of their interaction, more accurately mimicking the glycocalyx.^147^ Heteromultivalent binding dynamics^75,148^ of the cholera toxin^49,149^ and *Pseudomonas aeruginosa* lectin LecA,^147,150^ have been explored by changing the fluidity of the supporting membrane.

More recently, whole cells have been demonstrated as platforms which may offer enhanced functionality compared to conventional glycan arrays. This can be done in one of two ways. First, the surface expression of glycans can be manipulated by harnessing the mammalian cell’s own glycan synthetic machinery.^151^ This has only recently become feasible alongside the growing knowledge of the genes encoding glycosyltransferases,^152^ and the advent of CRISPR/Cas9 gene editing technology.^153^ There are now stable isogenic cell lines with defined glycosylation features^154^ which have been used to investigate the patterns of human Siglec glycan binding.^155^ This type of technology is also being applied more broadly to understanding cellular glycosylation,^156^ for example in characterizing the complex heterogeneity in mucin glycosylation,^157^ and differentiating the roles of glycosphingolipids, *N*-glycans and *O*-glycans in regulating leukocyte-endothelium adhesion.^158^ Second, whole cell surfaces can be functionalized to present multiple copies of specific glycans by post-translational chemoenzymatic methods. In this technology, libraries of mutant cells which express a small, homogeneous range of glycoforms are then treated with varying glycosyltransferase enzymes to install analogues of sialic acid and fucose.^159^ This approach allowed for the identification of high-affinity ligands for sialic-acid binding lectins,^159^ and the development of a cell-based influenza hemeagglutinin ligand array.^160^ Phage-based platforms, which display surface proteins which can be tagged with synthetic glycans, use DNA encoding to control the density of final carbohydrate presentation and to aid in the identification of ligands through deep sequencing.^161^ This technology has been further extended with on-phage enzymatic elaboration to produce complex *N*-glycans for arrays.^162^

While the density of glycan presentation is more easily controlled with the synthetic glycoconjugate scaffolds discussed here, it can be hard to replicate the heterogeneity present in biological systems without significant synthetic effort. By utilizing genetically engineered and chemoenzymatically altered whole cell platforms, it is simple to access controlled diversity in the glycans presented, although the more granular details of binding constants or cooperativity for individual interactions may not be possible to infer. A spectrum of technologies is available, ranging between precisely defined systems and fluid, whole-cell glycan arrays, and the ideal technology will depend greatly on the application and desired information.

## Multivalent Carbohydrate Vaccines

**Figure 5:**
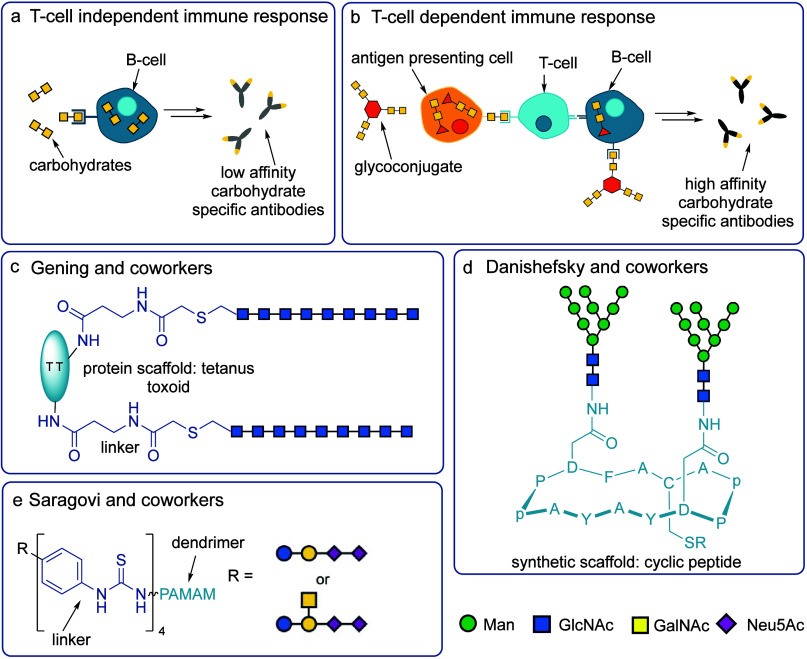
a) T-cell-independent immune response with carbohydrates. b) T-cell-dependent immune response using glycoconjugate. c) Semisynthetic antibacterial vaccine developed by Gening and co-workers.^170^ d) HIV vaccine candidate developed by Danishefsky and co-workers.^173^ e) Wholly synthetic anticancer vaccine candidate developed by Saragovi and co-workers.^178^

A therapeutic application of multivalent glycoconjugates that garners considerable research effort is vaccine development. Glycans are generally considered to be poorly immunogenic, failing to produce long lasting protection, particularly in infants and the elderly.^163−165^ This lack of persistent immunity is due to their recognition by the immune system primarily through B-cells, producing low-affinity carbohydrate specific antibodies (Figure 5a). To obtain long-term immunity, a response through T-cell recognition is required. In a key early discovery from Avery and Goebel, the immunogenicity of a carbohydrate could be improved by conjugation to a carrier, for example a protein. Uptake of the glycoconjugate antigen and its subsequent presentation allowed for a T-cell dependent immune response and production of high-affinity carbohydrate specific antibodies (Figure 5b).^166^

A list of current FDA approved glycoconjugate vaccines has been compiled recently.^163^ All vaccines listed, including those against diseases such as tetanus, diphtheria and typhoid fever, were obtained using cultivation of a pathogen containing the desired antigen. The major drawbacks with this method include variation of efficacy between batches, contaminants such as cell debris and safety concerns with large scale pathogen cultivation. Production of semisynthetic or fully synthetic vaccines could limit these problems, achieving consistent glycan attachment to a carrier and removing the need for large scale pathogen cultivation.

Both semisynthetic and fully synthetic vaccines consist of three main components: a synthetic carbohydrate antigen, a linker and a carrier.^167^ The key difference between these is the identity of the carrier, which is either from a natural source, such as a protein, or fully synthetic. An adjuvant, such as a mineral salt, an activating ligand or protein toxin, can be added to increase the uptake of the antigen. In semisynthetic vaccines, the adjuvant is part of vaccine formulation rather than a part of the vaccine structure, which is the case for fully synthetic vaccines.

The Cuban vaccine, Quimi-Hib, against the pneumonia and meningitis causing bacterium *H. influenzae*-B (Hib), became the first semisynthetic vaccine approved for human use and licensed by the WHO.^168^ A synthetic ribosyl-ribitol-phosphate oligosaccharide was used to replicate a key Hib capsular polysaccharide which, after conjugation to a protein carrier, was successfully able to incite an immune response against Hib in infants.^169^ The success of this vaccine has not yet been repeated, with no synthetic or semisynthetic glycoconjugate vaccines licensed since. Optimistically, numerous vaccine candidates against bacteria, both semisynthetic and fully synthetic, have entered preclinical and clinical trials.^164^ The majority are semisynthetic consisting of a synthetic glycan that is chemically linked to a protein, typically through a linker. An example by Gening and co-workers demonstrated the synthesis of a vaccine based on a GlcNAc-containing oligosaccharide conjugated using amide linkages to tetanus toxoid (Figure 5c).^170^ The glycoconjugate contained an average of 71 carbohydrate ligands on each toxoid. Preclinical trials in both mice and rabbit models found that the vaccine successfully protected against *Staphlyococcus aureus* skin abscesses and *E. coli* peritonitis. Multiple decasaccharide fragments conjugated onto carrier proteins were shown^171^ to elicit immune response against the fungal pathogen *Cryptococcus neoformans* in mice, leading to the production of opsonic antibodies and improving median survival.

The development of fully synthetic vaccines also garners considerable research effort, however no fully synthetic vaccines have been licensed.^164^ Current endeavors toward vaccines against human immunodeficiency virus (HIV) have identified a potential broadly neutralizing antibody that is carbohydrate specific, the 2G12 antibody.^172^ Mimics of the 2G12 epitope are being developed, consisting of d-mannose-dense structures. An example of such a mimic was synthesized by Danishefsky and co-workers consisting of branching 9Man2GlcNAc units attached to a cyclic peptide (Figure 5d).^173^ Cysteine residues within peptidic structures can be used to conjugate an adjuvant to the vaccine to improve its efficacy.^174^

A further application of multivalent glycoconjugates is toward the synthesis of anticancer vaccines and immunotherapy, an area of research that has been reviewed extensively in the past 15 years.^64,175−177^ These vaccines aim to target tumor-associated carbohydrate antigens (TACAs) in patients who either currently have cancer or are in remission. At present, no anticancer vaccines have the capability to work prior to infection and require tailoring to each patient. Some TACAs of interest are the GD2 and GD3 gangliosides which are glycolipids expressed at higher levels on the outer membrane of cancerous cells such as those associated with neuroblastomas and melanomas. Recently, Saragovi and co-workers developed two polyamidoamine (PAMAM) dendritic structures furnished with four copies of synthetic GD2 or GD3 carbohydrate moieties (Figure 5e).^178^ The glycans were synthesized chemoenzymatically with the final step using a GalNAc transferase to install a GalNAc residue selectively to the GD3 tetrasaccharide affording the GD2 pentasaccharide. Optimistically, during their studies, the dendrimers induced both an antibody response and activated T-cells, representing an important step in the development of anticancer vaccines.

Quimi-Hib has shown us that semisynthetic glycoconjugate vaccines are possible. A major bottleneck in the application of semisynthetic and fully synthetic vaccines is the large-scale synthesis of the desired glycans through increased manufacturing complexities and higher costs, when compared to obtaining glycans through pathogen cultivation,^168^ although as discussed above, this problem is becoming less of a roadblock with rapid advancements in automated solid-phase synthesis of carbohydrates and chemoenzymatic synthesis. Another roadblock for glycoconjugate vaccines is off-target effects and the high complexities of the target systems. This is particularly prevalent in TACA vaccine candidates where no vaccine has been successful in phase 3 trials.^175^ Increased understanding of the immune system and the tumor microenvironment through identification of new suitable glycan targets is a crucial step for the development of these vaccines.^179^

## Multivalent Glycoconjugate Therapeutics and Diagnostics

**Figure 6:**
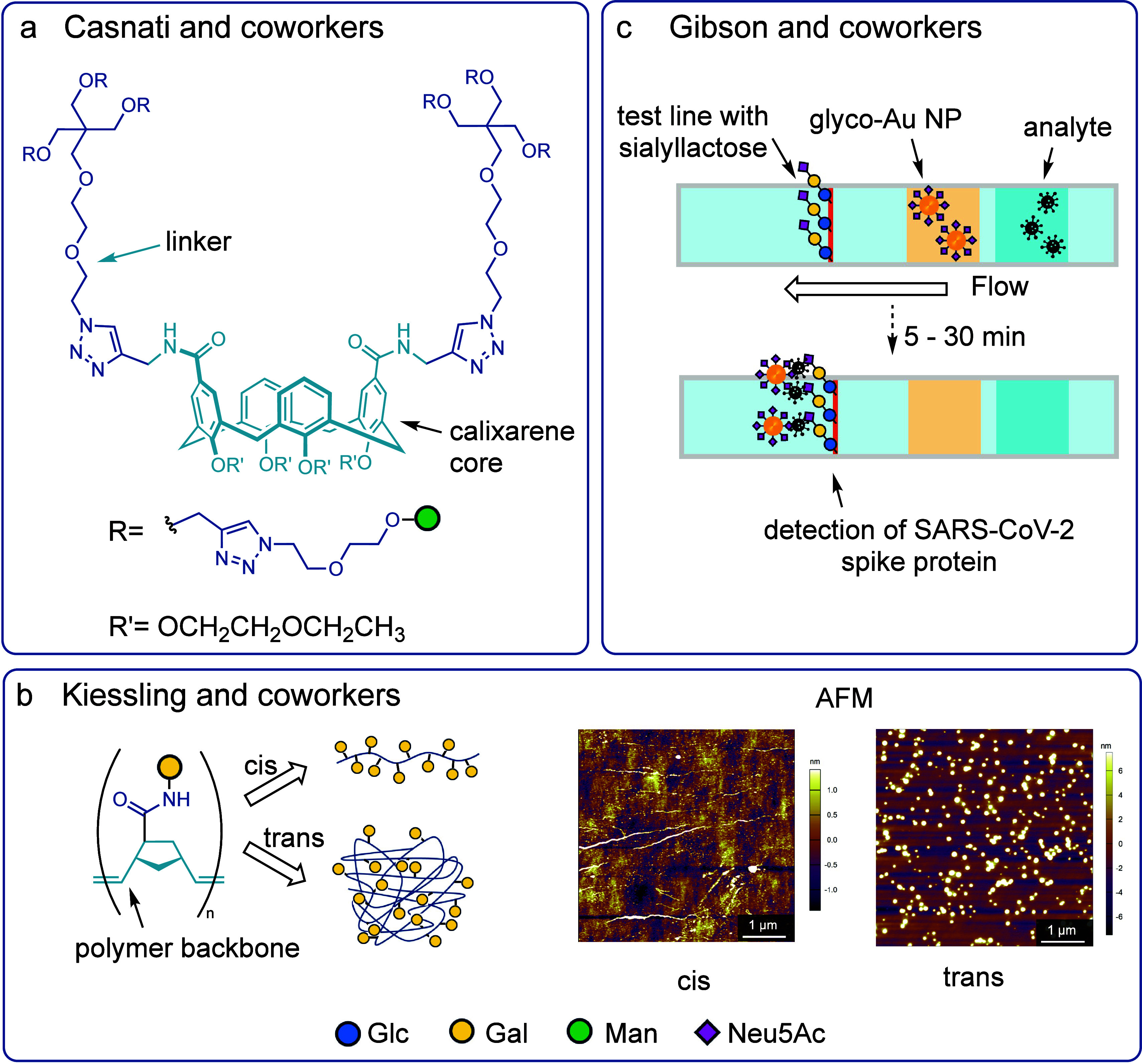
a) Antiadhesive therapies against *E. coli* strains developed by Casnati and co-workers.^211^ b) Mucin mimics synthesized by Kiessling and co-workers, partially reproduced from ref (218), copyright 2021 American Chemical Society. c) Lateral flow testing with Au nanoparticles developed by Gibson and co-workers.^227^

Multivalent glycoconjugates are being investigated for a variety of other therapeutic and diagnostic applications, including for lectin inhibition,^13,38,39^ enzyme inhibition,^180^ drug delivery,^181−183^ and imaging.^184,185^ The methods developed for achieving multivalency are as diverse as the end applications, with a few recent examples selected here to give an overview of the variety of multivalent structures and scope of the applications (Figure 6).

The macromolecular nature of multivalent glycoconjugates typically excludes them being considered “drug-like”, e.g., considering Lipinski’s rules.^186^ While likely to display poor oral bioavailability, multivalent glycoconjugates offer significant promise in the development of therapeutics and drug delivery systems. Multivalent glycoconjugates present excellent candidates for drug delivery,^181^ particularly in relation to cancer treatment, with many cancers displaying abherrent glycosylation patterns^187^ and altered carbohydrate receptor presentation. Tobacco mosaic viral capsids modified with d-mannose and d-lactose^188^ enabled targeted delivery of cisplatin to cancer cell lines displaying complementary cell-surface receptors, inducing apoptosis. Short interfering RNA (siRNA)^189^ is a promising and generalizable therapeutic strategy which “silences” the expression of a particular protein of interest by delivering a complementary RNA sequence. The strategy suffers, however, from high rates of extracellular degradation by RNases, and limited cellular uptake on account of the multiply negatively charged nature of siRNA.^190^ Cationic glycopolymer conjugates have been shown to allow internalization of siRNA,^191,192^ while in a similar approach a poly(galactaramidoamine) system^193^ has been commercialized as a reagent to enable DNA transfection to eukaryotic cells (Glycofect).

Given the rapid acceleration in antibiotic resistance,^194^ it is increasingly clear that we need to employ diverse strategies to control bacterial infections. Nonbactericidal agents which target the factors contributing to bacterial virulence^195^ are increasingly attractive, as they present reduced scope for the development of resistance.^194^ A seminal development in this area was the STARFISH dendrimer, synthesized by Bundle and co-workers,^33^ which was shown to neutralize the Shiga-like toxin produced by the highly virulent *E. coli* O157:H7 strain with subnanomolar inhibitory potency, representing an increase in *in vitro* activity of over a million-fold compared to the corresponding monovalent interaction. Using similar strategies of rational design, multivalent glycoconjugates have been demonstrated to inhibit the carbohydrate recognition domain of the structurally similar cholera toxin with picomolar inhibitory potency.^196^

Multivalent glycoconjugates have also been applied to the inhibition of carbohydrate-processing enzymes.^197,198^ Iminosugars are common synthetic candidates for both glycosidase and glycosyltransferase inhibition,^199^ and their incorporation onto multivalent scaffolds such as polymers^200^ and cyclodextrins^201^ have shown inhibition increases of 3–4 orders of magnitude. A particularly impressive example of potent multivalent enzymatic inhibition is a 9-valent pyrrolidinol-based mannose dendrimer, which inhibits the Jack Bean α-mannosidase protein with an IC50 of 95 nM and a 700-fold improvement over the constituent monomer.^202^ While this compound shows some selectivity for the Golgi mannosidase GMIIb over lysosomal mannosidase LManII, which could allow its development as a cancer therapeutic to target *N*-glycan processing pathways which lead to altered tumor glycosylation, a key difficulty in the clinical implementation of these compounds is selectively inhibiting one of many closely related glycosidases.^203^

Deficiency in the β-glucocerebrosidase enzyme (GCase), which is the underlying cause of the glycosphingolipid lysosomal storage disorder Gaucher disease, can be counterintuitively improved by the application of multivalent inhibitors in a technique called chaperone therapy.^204^ In this application, reversible multivalent inhibitors of the deficient enzyme at subinhibitory concentrations would bind and stabilize or induce proper folding, enhancing the residual catalytic activity before the enzyme could be degraded. For this purpose, the most potent inhibitor is not necessarily the best chaperone, and lower valency, more weakly binding multivalent systems induce higher GCase enzyme activity.^205,206^

Influenza A infection is dependent on the action of two carbohydrate-binding proteins^207^ on the surface of the viral capsid: a hemeagglutinin, which binds to sialyl-terminated cell-surface glycans to infect cells, and a neuraminidase which cleaves sialic acid glycosides on the surface of infected cells to facilitate release of viral progeny. Multivalent glycoconjugates with complementary lectin recognition motifs present a promising strategy for the development of anti-influenza therapeutics. Polyglycerols decorated with 6′-sialyllactose and zanamivir,^208^ a neuraminidase inhibitor, have been designed to enable simultaneous targeting of hemagglutinin and neuraminidase. Hemagglutination inhibition data suggests increased adhesion of heteromultivalent polymers compared to their homomultivalent analogues onto viral capsids. In human lung *ex vivo* studies, the heteromultivalent polymer was also observed to outperform zanamivir, along with the homomultivalent polymers, even when applied together, demonstrating the synergistic effect of heteromultivalency.

The multivalent presentation of carbohydrate ligands on a macromolecular scaffold also presents opportunities for the design of antiadhesive agents, which act to prevent the adhesion of bacteria to cellular surfaces in the initial stages of infection. For example, uropathogenic *E. coli* strains are a principal cause of urinary tract infections which can lead to chronic disease and complications though the development of biofilms, and their adhesion is often mediated through interaction of the FimH protein with a mannosylated cell surface glycoprotein,^209^ in a “catch-bond”. The initial transient interaction between the mannoside and FimH has fast binding and release kinetics (*t*_1/2_ ≈ 12 ms), allowing for bacterial motility along the bladder epithelium. However, the introduction of shear force for example by urination, induces an allosteric change in protein conformation which slows down the disassociation rate by 100,000-fold, preventing the bacterium being cleared from the body.^210^ Dendrimers displaying a calixarene core furnished with d-mannose (Figure 6a) were demonstrated to bind to uropathogenic *E. coli*, with STD-NMR experiments confirming adhesion was mediated through interaction with FimH.^211^ Mannosylated dendrimers also incorporating an aromatic aglycone unit^212^ have been shown to display enhanced affinity for FimH with *K*_d_ = 0.45 nM, and were shown to inhibit the binding of *E. coli* to erythrocytes *in vitro*.

Biofilm formation in *P. aeruginosa* is assisted by the action of a galactosyl-binding lectin, LecA, and a fucosyl-binding lectin, LecB.^10^ Dendrimers decorated with d-galactose,^213^d-fucose,^214^ or a combination of the two sugars^215^ have been shown to bind to LecA/LecB with *K*_d_’s in the nanomolar range, and disrupt biofilm formation with MICs as low as 10 μM. When combined with conventional antibiotic therapies such as tobramycin, heteroglycodendrimers enabled effective dispersion of biofilms at submicromolar concentrations of either therapeutic, demonstrating potential for the application of multivalent glycoconjugates within current therapeutic treatment regimes. Fucosylated and galactosylated calix[4]arene-based glycoclusters were shown by ITC to recognize LecB/LecA with nanomolar affinities,^216,217^ and to significantly suppress biofilm formation in *P. aeruginosa* without suppressing bacterial growth,^216^ demonstrating an antivirulence mechanism. These glycoclusters were also shown^216^ to inhibit *P. aeruginosa* adhesion to human epithelial cells, and to protect against *P. aeruginosa* induced lung injury in a mouse pulmonary infection model, demonstrating that multivalent binding to disease associated lectins can decrease bacterial virulence and offering promise for the use of multivalent glycoconjugates as anti-infective agents.

Mimicking natural structures synthetically is an important step in the development of new therapeutics. Mucin, a densely glycosylated polypeptide, is the primary component of mucus and provides an important barrier for cells against microbial infections and toxins. Kiessling *et al* recently investigated glycopolymer based mucin mimics using different catalysts for a ring-opening metathesis polymerization (ROMP) to obtain *cis*- and *trans*-orientated polymer backbones (Figure 6b).^218^ These d-galactose furnished polymers presented distinct morphologies, with the *cis*-alkene systems better mimicking the extended brush-like structure of natural mucins, providing a template for synthetic mucin substitutes in future experiments.^219^

Interest in the use of glyconanoparticles as diagnostic tools has also been garnering attention.^184^ Aberrant protein glycosylation patterns are well-known to correlate with disease states such as cancer.^220^ For example, the prostate specific antigen protein (PSA) is clinical biomarker for prostate cancer, but disease severity cannot be inferred from its presence, leading to overtreatment of indolent cases.^221^ However, branched, multivalent α-2,3-linked sialic acid terminal residues on PSA have been correlated with aggressive prostate cancer in multiple studies.^222,223^ To this end, a proof of concept, high throughput assay has been developed that uses antibody-coated surfaces to extract PSA from a mixture, followed by lectin-functionalized gold nanoparticles which can distinguish glycosylated and nonglycosylated forms.^224^

Colorimetric and fluorometric approaches to detect disease markers are attractive from a point-of-care context on account of their rapid throughput, and typically high-sensitivity. With these applications in mind, tetraphenylethylene scaffolds have been decorated with multiple copies of carbohydrates that bind to the bacterial toxins^225^ or viral surface proteins.^226^ Multivalent binding to the target analyte results in aggregation-induced emission, presenting a modular platform for the construction of fluorescence-based sensors. Gibson and co-workers have developed a detection system employing a neuraminic acid as a ligand for the spike glycoprotein present on the surface of SARS-CoV-2 (Figure 6c).^227^ α-*N*-Acetyl neuraminic acid was demonstrated to bind to the spike protein via STD-NMR experiments, and was attached to chain termini of poly(*N*-hydroxyethyl acrylamide), before immobilization onto gold nanoparticles (AuNPs). These multivalent glycoconjugates were combined with the analyte within the mobile phase of lateral flow assays. α-2,6′-Sialyllactose immobilized onto the test line provided a secondary ligand to capture AuNP-labeled proteins, enabling clear detection of the spike protein in under 30 min and displaying selectivity for SARS-CoV-2 compared to the spike protein of SARS-CoV-1, another zoonotic coronavirus. Given the prevalence of glycan recognition within many diseases, this approach presents a platform technology that could easily be adapted to make diagnostics for a range of bacterial or viral pathogens and exploiting multivalent recognition.

As discussed above within the context of glycan arrays, there is a move toward creating biologically engineered whole-cell systems for the specific display of multivalent glycoconjugates for therapeutic and diagnostic applications. Early work used chemoenzymatic glycocalyx editing to produce cells with specific glycosylation patterns, for example using fucosyltransferase to install E-selectin ligands onto the cell surface to enhance engraftment and trafficking of stromal cells^228^ and cord blood cells.^229^ More recently, a similar chemoenzymatic glycan modification strategy has been used to probe the link between glycans and membrane receptor signaling,^230^ and to regulate stem cell proliferation.^231^ The development of this type of live-cell glycocalyx engineering is paving the road toward glycotherapies in numerous areas including cancer and autoimmune diseases.^232^

## Outlook

The progress made in complex glycan synthesis and characterization over the past 20 years has brought about a “golden age” for glycobiology. Automated synthesis, chemoenzymatic methods, and the huge diversity in scaffolds available means chemists can synthesize multivalent carbohydrates which more and more closely approximate the complexity and diversity seen in nature. This is complemented by the tools that have been developed for the characterization of complex systems, including mass spectrometry and chromatography techniques, as well as a host of chemical biology and bioinformatics techniques which can more accurately predict the natural systems we are seeking to emulate. With these tools and techniques to hand, there remains many challenges and opportunities for synthetic chemists to expand our understanding of glycobiology.

Heteromultivalent systems present an area which deserves increased attention, both for our fundamental understanding of interactions with the glycocalyx in nature, and for the benefit of therapeutic and diagnostic design. There is evidence to suggest that secondary binding effects could have different entropic and enthalpic contributions than their corresponding homomultivalent systems,^80,233^ allowing weakly binding ligands within fluid membranes to play an important role in heterovalent binding.^149,150^ This effect may contribute to complex regulation of signaling in biological systems, further lending weight to the idea that multivalency allows for a dynamic response range, rather than simple on/off switches.^75^ In model studies^234^ investigating the interactions of synthetic glycopolymers and a mannose binding lectin, Con A, heteroglycopolymers bearing α-mannose and nonbinding β-glucose or β-galactose units, were shown to exhibit an approximately 5-fold increase in binding affinities compared to polymers decorated only with α-mannose. Similarly, glyconanoparticles^235^ assembled using glycopolymers decorated with both d-mannose and d-galactose were found to enable higher endocytosis efficiency than glyconanoparticles constructed using mixtures of d-mannose-decorated and d-galactose-decorated glycopolymers. Recent applied examples of heteromultivalent liposomes have also shown more specific protein targeting^82^ and increased accumulation as drug-delivery agents^83^ as a result of incorporating secondary binders. Incorporating multiple binding motifs has the opportunity to unlock a new generation of therapies with improved specificity, and could be implemented as a routine phase of investigation in their design.

The fluid, three-dimensional presentation of a glycan within a dynamic surface is a factor deserving increased consideration in the design of multivalent systems. Multivalent interactions occur over large surface areas, and the glycocalyx is thought to regulate membrane shape by exerting forces that bend the plasma membrane in high-density regions,^236^ and on a smaller scale, there is evidence that GM1 clusters cause membrane perturbation in synthetic liposomes.^237^ The recent development of fluid membrane glycoconjugates will allow for the further investigation of these fundamental cellular processes, which are posited to be not merely a byproduct of glycocalyx arrangement but also a key cellular signaling mechanism that determines shape and migration from cellular to tissue length scales.^238^

Finally, multivalency produces opportunity for aggregates as proteins and multivalent glycans interact with one another through networks. There is evidence that multivalent ligands and receptors can aggregate and become kinetically trapped as a result of a phase transition.^239^ This process can occur beneficially in nature—protein granules formed through nonspecific multivalent interactions have unique material properties that are implicated in functions as diverse as cellular filtration, and sensing and memory.^240^ There also appears to be a key role for clustering in the localization of enzymes, reagents and cofactors at high local concentrations in granule “factories” which allow significant increases in the output of key transformations.^241^ However, aggregation can also be detrimental, and it appears that the progression of aggregates to irreversible, nonequilibrium structures tends to be more associated with disease pathology,^242^ with evidence that *N-*glycans play an important role in protein assembly in different disease states.^243^ Modeling of these larger systems through defined synthetic multivalent assemblies is likely to be an interesting and highly fruitful avenue of investigation.

The ability to synthesize mimics of the multivalent glycoconjugates we see in nature affords the potential to understand their behavior on a fundamental level. Further to these examples, there will be a plethora of new biological questions, as well as applications in diagnostics and therapeutics, which are fertile ground for synthetic chemists who can increasingly make systems that reproduce the complexity and diversity seen in nature.
